# Complete mitochondrial genome of the introduced Indian walking stick *Carausius morosus* (Lonchodidae, Insecta) from California

**DOI:** 10.1128/mra.00321-24

**Published:** 2024-05-31

**Authors:** Aiden Clarke, Alice Trujillo, Andres Mandujano, Angelica G. Fernandez, Aniyah Chambers, Areli Ruiz Nunez, Audri Contreras, Benny Cuevas, Caitlin Collins, Christian B. Trujillo, Claudia L. Dominguez-Trejo, Danilo E. Bustamante, Eduardo Pantoja-Garcia, Elizabeth Anguiano, Emily D. Alcaraz, Felipe Rodriguez, Flavio C. Mora, Froylan Tinoco Rivera, Gladys Cabrera Luis, Hailey B. Nava, Henry N. Huynh, Javier C. Diaz, Jeffery R. Hughey, Jenny Do, Jeriel S. Sevilla, Jessica C. Llaja, Jessica Lopez, Jesus Rosas, Jhordy Perez, Johann E. Oyola, Jois V. Carrion, Joni J. Black, Jorge F. Chavez, José I. Barboza, Juan Pablo Rodriguez Cortes, Konnor L. Barrett, Lacey E. Prescott, Layla Alvarez, Lizbet Merino Juarez, Maria J. Velasquez-Moreno, Mariah I. Marquez-Gonzalez, Mariana Aguirre Linares, Maricela Chavez-Huigo, Martha S. Calderon, Mateo Brambila, Maximiliano Villa, Mia J. Windham, Michael Perez, Natalie Trujillo, Pearl Chenevert, Phoebe Lewis, Pilar Guiop, Reema Y. Mubarz, Roberto Garcia Velazquez, Rosmery Y. Ayala-Tocto, Samantha Santos, Samia L. J. Fernandez-Güimac, Sandra R. Zalasar, Smith E. Aguilar-Trauco, Soledad Duran, Stephanie Solis, Steven L. Meza, Taym Al-Zuhairi, Victor M. Padilla, Yadhira M. Olano, Yareli Alfaro Maldonado, on behalf of Hartnell College Genomics Group

**Affiliations:** 1 Division of Mathematics, Science, and Engineering, Hartnell College, Salinas, California, USA; 2 Instituto de Investigación para el Desarrollo Sustentable de Ceja de Selva (INDES-CES), Universidad Nacional Toribio Rodríguez de Mendoza, Chachapoyas, Amazonas, Peru; 3 Instituto de Investigación en Ingeniería Ambiental (INAM), Facultad de Ingeniería Civil y Ambiental (FICIAM), Universidad Nacional Toribio Rodríguez de Mendoza, Chachapoyas, Amazonas, Peru; University of Maryland School of Medicine, Baltimore, Maryland, USA

**Keywords:** mitogenome, non-native, Phasmatodea

## Abstract

We present the complete mitochondrial genome of *Carausius morosus* from Salinas, CA. The mitochondrial genome of *C. morosus* is circular, AT rich (78.1%), and 16,671 bp in length. It consists of 13 protein-coding, 22 transfer RNA, and 2 ribosomal RNA genes and is identical in gene content to *Carausius* sp.

## ANNOUNCEMENT

*Carausius morosus* Brunner Von Wattenwyl 1907, the Indian walking stick, is a large polyphagous species originally described from specimens from Shembagonor and Trichinopoly, Madura Province, southern India (1, 2). It was introduced to Australia, Azores, Madagascar, Madeira, South Africa, United Kingdom, and United States (3), likely due to the escaping of captive *C. morosus* and the accidental discarding of eggs (4). *Carausius morosus* was first reported from San Diego County, CA, in 1991 and has since spread to 10 counties where it is a pest on ornamental plants (4, 5). According to the California Department of Food and Agriculture, *C. morosus* has a high environmental impact score but a low overall pest rating (6). To contribute to the phylogenomics of Phasmatodea and the bioinformatics of *C. morosus*, the complete mitochondrial genome of *C. morosus* from California was assembled and characterized.

**Fig 1:**
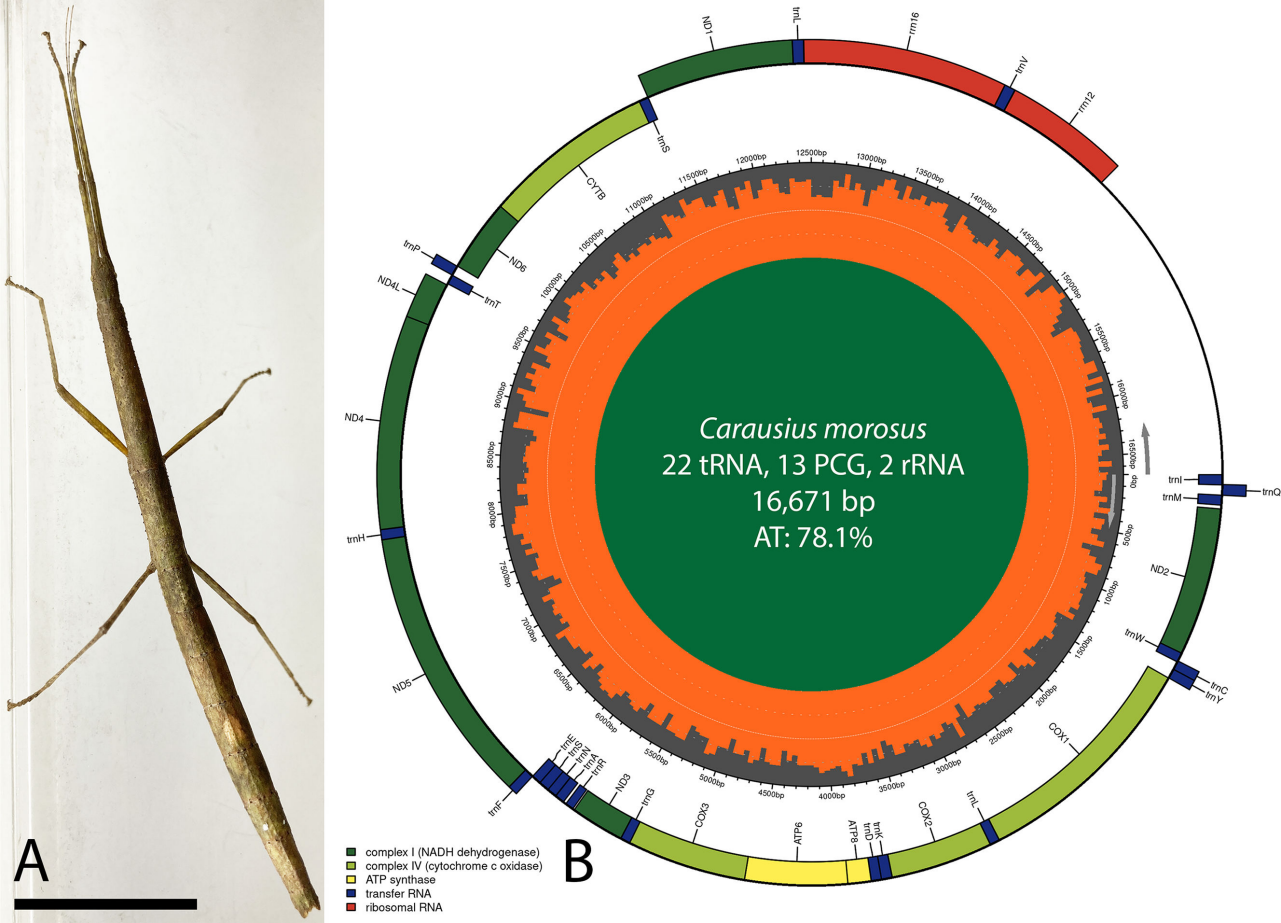
*Carausius morosus* analyzed in this study (**A**) and the complete mitochondrial genome map of *C. morosus* (**B**). (**A**) Specimen of *C. morosus* genome sequenced. Scale bar = 2 cm. (**B**) The genome was annotated using Geneious Prime and mapped with Chloroplot 0.2.4 (13). The innermost ring displays the AT content in Saffron color and the direction of transcription, as indicated by the arrows. The final ring displays the genes. Genes transcribed clockwise are on the inside, while counterclockwise transcriptions are positioned on the outside. The color coding corresponds to genes of different groups as listed in the key in the bottom left. PCG, protein-coding genes.

The specimen of *C. morosus* analyzed here (Fig. 1A) was collected from a coffee house stockroom in Salinas, Monterey County, CA (36.7000 N 121.6201 W), voucher number Hartnell College Collection #272 (Salinas, CA). The DNA was extracted using the DNeasy Blood and Tissue Kit (Qiagen) following a previously published protocol (7). The 150-bp paired-end library was constructed with the KAPA HyperPlus Kit (Roche) and sequenced on an Illumina NovaSeq 6000 (Illumina Inc.). The sequencing generated 12,826,468 reads that were filtered using the default BBDuk 1.0 (8) settings in Geneious Prime 2019.1.3 (Biomatters Limited). The mitochondrial genome was assembled *de novo* using a kmer ≥ 79 with MEGAHIT 1.2.9 (9). The assembly yielded 97,733 contigs with an *N*_50_ of 469 and GC content of 37.5%. A single *C. morosus* mitochondrial contig with 119-bp overlapping ends and 49.5× coverage was identified by a two-way Nucleotide BLAST 2.15.0+ search using the default settings (10). The overlapping ends were removed manually, and the mitochondrial genome start position was adjusted to correspond with *Carausius* sp. (GenBank accession number OQ682524). The annotation was performed in Geneious Prime designating the *Carausius* sp. mitochondrial genome as the reference with a similarity index of 65%. Gene start and stop positions were confirmed by comparison to 56 complete mitochondrial genomes of Phasmatodea in GenBank and using standard, universally accepted initiation and termination codons as defined in the invertebrate mitochondrial genetic code (11, 12). Nucleotide identities were calculated by BLAST search using the default settings.

The complete circular mitochondrial genome of *C. morosus* is 16,671 bp in length and has a AT bias of 78.1%. Genome content and structure are identical to *Carausius* sp. (14). The genome contains 37 genes including 13 protein-coding, 22 tRNA, and 2 rRNA genes (Fig. 1B). Five of the protein-coding genes initiate with ATA (ATP6, ATP8, COX2, ND2, and ND4L), three with ATT (ND3, ND5, and ND6) and ATG (COX1, COX3, and CYTB), and one with GTG (ND4) and TTG (ND1) (Table 1). The TAA termination codon is found in all genes except CYTB (TAG) and COX2 [T, stop codon is completed by the addition of 3′ A residues to the mRNA, as is common in animal mitochondrial genomes (15, 16)]. The entire mitochondrial genome sequence of *C. morosus* is 80.85% similar to the genome of *Carausius* sp. from China and 100% and 99.3% similar to the gene sequences COX1/COX2 and 16S rRNA, respectively, of *C. morosus* from India.

**TABLE 1:** Mitochondrial genome content, organization, and codon information of *Carausius morosus*

Gene	Type	Minimum nucleotide position	Maximum nucleotide position	Length	Start codon	Stop codon	Direction
tRNA-Ile	tRNA	1	67	67	–^*a*^	–	Forward
tRNA-Gln	tRNA	65	134	70	–	–	Reverse
tRNA-Met	tRNA	134	198	65	–	–	Forward
ND2	CDS	223	1,194	985	ATA	TAA	Forward
tRNA-Trp	tRNA	1,193	1,256	64	–	–	Forward
tRNA-Cys	tRNA	1,249	1,310	62	–	–	Reverse
tRNA-Tyr	tRNA	1,310	1,373	64	–	–	Reverse
COX1	CDS	1,375	2,908	1534	ATG	TAA	Forward
tRNA-Leu	tRNA	2,909	2,973	65	–	–	Forward
COX2	CDS	2,974	3,643	670	ATA	T	Forward
tRNA-Lys	tRNA	3,644	3,714	71	–	–	Forward
tRNA-Asp	tRNA	3,714	3,780	67	–	–	Forward
ATP8	CDS	3,781	3,936	156	ATA	TAA	Forward
ATP6	CDS	3,933	4,604	672	ATA	TAA	Forward
COX3	CDS	4,604	5,386	783	ATG	TAA	Forward
tRNA-Gly	tRNA	5,386	5,449	64	–	–	Forward
ND3	CDS	5,450	5,803	354	ATT	TAA	Forward
tRNA-Arg	tRNA	5,814	5,869	56	–	–	Forward
tRNA-Ala	tRNA	5,889	5,952	64	–	–	Forward
tRNA-Asn	tRNA	5,953	6,018	66	–	–	Forward
tRNA-Ser	tRNA	6,017	6,086	70	–	–	Forward
tRNA-Glu	tRNA	6,086	6,148	63	–	–	Forward
tRNA-Phe	tRNA	6,149	6,209	61	–	–	Reverse
ND5	CDS	6,210	7,934	1721	ATT	TAA	Reverse
tRNA-His	tRNA	7,935	7,997	63	–	–	Reverse
ND4	CDS	7,998	9,329	1332	GTG	TAA	Reverse
ND4L	CDS	9,323	9,607	285	ATA	TAA	Reverse
tRNA-Thr	tRNA	9,616	9,678	63	–	–	Forward
tRNA-Pro	tRNA	9,679	9,744	66	–	–	Reverse
ND6	CDS	9,746	10,222	478	ATT	TAA	Forward
CYTB	CDS	10,222	11,355	1134	ATG	TAG	Forward
tRNA-Ser	tRNA	11,354	11,417	64	–	–	Forward
ND1	CDS	11,416	12,384	969	TTG	TAA	Reverse
tRNA-Leu	tRNA	12,385	12,453	69	–	–	Reverse
16S rRNA	rRNA	12,455	13,726	1272	–	–	Reverse
tRNA-Val	tRNA	13,727	13,794	68	–	–	Reverse
12S rRNA	rRNA	13796	14578	783	–	–	Reverse

^*a*^ –, not applicable.

## Data Availability

The complete mitochondrial genome sequence of *Carausius morosus* is available in GenBank under accession number PP230539. The associated BioProject, SRA, and BioSample numbers are PRJNA1089237, SRS20783361, and SAMN40533979, respectively. The mitochondrial genome referenced in the text is *Carausius* sp. GenBank accession number OQ682524.
